# The effect of chronotropic incompetence on physiologic responses during progressive exercise in people with Parkinson’s disease

**DOI:** 10.1007/s00421-024-05492-5

**Published:** 2024-04-29

**Authors:** Tone Ricardo Benevides Panassollo, Sue Lord, Usman Rashid, Denise Taylor, Grant Mawston

**Affiliations:** 1 School of Clinical Sciences, Auckland University of Technology, 90 Akoranga Drive, Northcote, 0627 Auckland, New Zealand; 2 Centre for Chiropractic Research, New Zealand College of Chiropractic, 6 Harrison Road, Mount Wellington, 1060 Auckland, New Zealand

**Keywords:** Heart rate, Oxygen consumption, Cardiopulmonary exercise test, Exercise prescription

## Abstract

**Purpose:**

Heart rate (HR) response is likely to vary in people with Parkinson’s disease (PD), particularly for those with chronotropic incompetence (CI). This study explores the impact of CI on HR and metabolic responses during cardiopulmonary exercise test (CPET) in people with PD, and its implications for exercise intensity prescription.

**Methods:**

Twenty-eight participants with mild PD and seventeen healthy controls underwent CPET to identify the presence or absence of CI. HR and metabolic responses were measured at submaximal (first (VT1) and second (VT2) ventilatory thresholds), and at peak exercise. Main outcome measures were HR, oxygen consumption (*V*O_2_), and changes in HR responses (HR/WR slope) to an increase in exercise demand.

**Results:**

CI was present in 13 (46%) PD participants (PDCI), who during CPET, exhibited blunted HR responses compared to controls and PD non-CI beyond 60% of maximal workload (*p* ≤ 0.05). PDCI presented a significantly lower HR at VT2, and peak exercise compared to PD non-CI and controls (*p* ≤ 0.001). *V*O_2_ was significantly lower in PDCI than PD non-CI and controls at VT2 (*p* = 0.003 and *p* = 0.036, respectively) and at peak exercise (*p* = 0.001 and *p* = 0.023, respectively).

**Conclusion:**

Although poorly understood, the presence of CI in PD and its effect on HR and metabolic responses during incremental exercise is significant and important to consider when programming aerobic exercises.

## Introduction

Parkinson’s disease (PD) is the second most common neurodegenerative disorder, affecting approximately 6 million people worldwide (Rocca 2018). The cardinal signs of PD are tremor and motor disturbance due to bradykinesia which affects gait, postural control, and motor function (Draoui et al. 2020). Non-motor symptoms such as cognitive impairment, fatigue, and impaired autonomic nervous system (ANS) are also common and debilitating (Santos-Garcia & de la Fuente-Fernandez 2013). Levels of physical activity are low in people with PD even in early disease (Lord et al. 2013) which in turn increases the risk of developing comorbidities (Liguori et al. 2022). While there is no cure for the disease, dopaminergic replacement therapies and structured exercise programs are the mainstay of management and are tailored to meet individual needs as the disease progresses (Fox et al. 2018; Gamborg et al. 2022).

ANS symptomatology is commonly reported in PD during the prodromal phase and as the disease progresses (Stankovic et al. 2019). Chronotropic incompetence (CI), defined as the inability to increase heart rate (HR) in response to an increase in demand during exercise testing despite attaining maximum effort (Brubaker and Kitzman 2011), is a feature associated with ANS dysfunction in PD (Palma et al. 2013). The prevalence of CI in PD is high with estimates around 50% (Kanegusuku et al. 2016; Penko et al. 2021) and it can be evident prior to disease onset (Palma et al. 2013; Palma and Kaufmann 2014). CI is often undetected in clinical practice and neglected in PD studies, despite its association with cardiovascular diseases (CVD), increased risk of mortality, and poor aerobic capacity reported in other conditions (Brubaker and Kitzman 2011; Herbsleb et al. 2018; Myers et al. 2007).

Although recent evidence suggests reduced aerobic capacity at submaximal and maximal intensity in people with PD (Kanegusuku et al. 2016; Mavrommati et al. 2017; Penko et al. 2021), this is not a universal finding (DiFrancisco-Donoghue et al. 2009; Protas et al. 1996). These studies mostly recruited sedentary and low physically active PD participants and did not stratify for presence of CI. In addition, there is limited knowledge regarding HR and metabolic responses in people with PD with CI using key submaximal physiologic markers (first (VT1) and second (VT2) ventilatory thresholds). Understanding these responses is crucial as they represent the physiologic points at which exercise intensity transitions from light to moderate (VT1) and from moderate to high (VT2) exercise intensities (Anselmi et al. 2021; Mezzani et al. 2013). Incorporating these thresholds into aerobic exercise training has shown greater effectiveness compared to traditional intensity measures (Anselmi et al. 2021; Meyler et al. 2021; Pymer et al. 2020).

Scant data are currently available on HR and metabolic response at submaximal (VT1 and VT2) and maximal intensities in people with PD with CI. This exploratory study aims to examine HR and metabolic responses during cardiopulmonary exercise test (CPET) in people with PD with and without CI to understand its effect on key physiologic markers and thereby enhance precision in setting exercise intensity parameters for aerobic exercise in this population.

## Methods

### Participants

People aged 45–75 years old with a clinical diagnosis of PD and age-matched controls were recruited via community-based organizations. Exclusion criteria included: the use of deep brain stimulation and the presence of neurodegenerative diseases other than PD for the PD group. For both groups, the exclusion criteria included the use of beta-blockers medication, having had a heart attack within the last year, having cardiomyopathy (such as dilated cardiomyopathy and hypertrophic cardiomyopathy), chronic obstructive pulmonary disease, or other conditions that contraindicated maximal exercise testing (Liguori et al. 2022). All participants signed an informed consent form approved by the Health and Disability Ethics Committees (HDEC) and the Auckland University of Technology Ethics Committees (AUTEC) adhering to the Declaration of Helsinki (Williams 2008). PD participants were tested “on” medication (approximately 1 h after medication). The International Physical Activity Questionnaire for the Elderly (IPAQ-E) was used to record each participant’s self-reported level of physical activity in the last 7 days prior to assessment.

### Cardiopulmonary exercise test (CPET)

CPET was performed on an electronically braked cycle ergometer (Daum, premium 8i, Germany) in a temperature-controlled room (19–20 °C). Participants were asked to refrain from exercise within 24 h of testing, to abstain from alcohol and caffeine for 12 h, and to avoid a large meal 3 h prior to testing. The CPET ramp protocol started with 3 min rest on the bike followed by a 3 min warm-up cycling at 60 revolutions per minute (rpm) with a workload (WR) of 20 watts. Resistance then increased incrementally by 15 watts per minute and the test terminated when recommendations from the American College of Sports and Medicine (ACSM) were met (Liguori et al. 2022), or when the participant could no longer cycle above 60 rpm. This was followed by a 5-min recovery phase cycling at a comfortable pace (20 watts). HR was continuously monitored throughout CPET via a 12-lead electrocardiogram (ECG, Customed Cardio 300, Germany).

## Outcomes

### Heart-rate response

Raw data were exported into NI LabView software 2021 (National Instruments) for further analysis. Exported data were interpolated and filtered using a moving average of five points (Cheng et al. 2008) for determination of changes in HR (HR/WR slope) from 30 to 100% of maximum WR (WRmax), maximum HR (HRmax), and HR recovery. HR/WR slope was measured using HR at intervals of 10% WR from 30 to 100% WRmax. Intensities below 30% WRmax were not included because at 20% WRmax two participants attained an intensity below 20 watts, which represented the warm-up resistance (Savonen et al. 2006). In this study, HRmax reflected the highest HR attained during CPET. HR recovery was calculated using final HR at exercise completion (HR at 100% WRmax) minus HR at the first (HR1min) and second minute (HR2min) of the recovery phase (Buchheit et al. 2007).

CI has often been identified using an arbitrary threshold of 85% of age-predicted maximum HR (Penko et al. 2021). However, this measure can be influenced by factors such as resting HR (HRrest) and aerobic fitness. Therefore, we used the chronotropic index equation: [(HRmax – HRrest)/(220 – age – HRrest)], which accounts for resting heart rate, to determine CI, with a result below 0.8 indicating CI (Lauer et al. 2005; von Scheidt et al. 2019). HRrest was obtained from an averaged 10 s ECG recorded before CPET in a supine position.

### Metabolic response

Metabolic data were collected breath by breath via a gas analyser (MetaLyzer 3B; Cortex Biophysik, Germany), throughout the CPET and during recovery. Prior to testing, the equipment was calibrated following the manufacturer’s instructions. MetaSoft Studio^©^ software (version 5.13.0 SR2) was used to filter data using a 20 s moving time interval average (Robergs et al. 2010). Thus, the highest average oxygen uptake over 30 s was considered the peak oxygen consumption (*V*O_2_ peak). VT1 and VT2 were determined independently by two experienced CPET clinicians using standardize guidelines that have been shown to have high reliability (Franssen et al. 2022). Criteria for maximum effort included a respiratory exchange ratio (RER) of ≥ 1.10 and a rating of perceived exertion (RPE) above 18 (Liguori et al. 2022; Robergs et al. 2010). Systolic (SBP) and diastolic (DBP) blood pressure were measured manually at rest before the test, every 2 min during the test, immediately afterward, and during the 5 min recovery phase of CPET. RPE was assessed using the Borg 6–20 scale at each 1 min through the incremental phase and at the end of the test (Borg 1982). Participants were familiarized with the Borg scale prior to testing.

## Statistical analysis

Demographic data were analyzed using ANOVA to observe differences across groups. Post hoc tests using Tukey’s Honestly Significant Difference were conducted to identify specific variations between groups. In the case of PD-specific variables such as years living with PD, levodopa equivalent, and UPDRS, T-tests were used to compare means, assuming independent observations and equal variances.

For CPET data, linear regression and mixed linear regression were used to evaluate the relationship between outcomes and independent variables. All models were adjusted for gender, age, height, years living with PD, levodopa dosage, IPAQ_sit, IPAQ_walk, IPAQ_mod, and IPAQ_vig. Body mass index (BMI) was included in the models for HR, %HR, RPE, DBP, SBP, and *V*O_2_/HR. Linear regression analysis was used for variables measured at a single time point (RER, SBP, DPB, *V*O_2_/WR slope, and VO_2_/HR) and included Group as the categorical variable. Mixed linear regression analysis was used for variables measured at multiple time points (HR, %HR, WR/Kg, *V*O_2_, % *V*O_2_, and RPE) and included a full interaction between Group and Time as the categorical variables. Mixed linear regression analysis was also used for HR/WR slope and included a full interaction between Group as a categorical variable and %WR as a continuous variable. We evaluated the suitability of fitting straight lines or curvilinear natural splines using Akaike’s information criterion. We also fitted correlated participant-wise random intercepts and slopes across %WR.

Assumptions of normality and homogeneity of variance for model residuals were evaluated with QQ plots and fitted-values VS residuals plots. Multicollinearity was evaluated with a variance inflation factor (VIF) and variables with VIF greater than 10 were excluded from the models. Means and slopes estimated from the models were reported along with 95% confidence intervals. The threshold for statistical significance was set at 0.05. Data were analyzed in R environment for statistical computing (Bates et al. 2015).

The sample size was determined for t-tests (GPower 3.1.9.7) with a 0.05 significance level and 80% power. Based on data from a previous study (Kanegusuku et al. 2016), 8 participants per group were required for the primary outcome (HRmax), while 28 participants per group were needed for the secondary outcome (*V*O_2_ peak). To ensure adequate representation, a minimum sample size of 16 PD was set for the primary outcome, based on an estimated prevalence of CI in PD of around 50%. However, due to COVID-19, budget, and time constraints, the required sample size of 56 individuals with PD for the secondary outcome could not be achieved.

## Results

### Study-group characteristics

Thirty-two participants with mild PD (Hoehn and Yahr (H&Y) I-III) and eighteen healthy controls were initially recruited and consented. Five participants were excluded due to a history of CVD (2 PD and 1 control), hip replacement (1 PD) and for personal reasons (1 PD). Thus, a total of 28 PD and 17 heath control participants were included, with the demographic data are presented in Table 1*.* CI was identified in 13 (46%) PD participants who were included in the PDCI group. All other participants were included in the PD non-CI (*n* 15) or control group (*n* 17).

**Table 1:** Demographic data

	PDCI (*n* = 13)	PD non-CI (*n* = 15)	Control (*n* = 17)
Age; years	64 ± 6.01 [55, 74]	62 ± 5.56 [52, 70]	62 ± 6.27 [52, 70]
Males (%)	9 (69%)	8 (53%)	9 (53%)
Height; cm	174 ± 7.51 [162, 188]	172 ± 10.75 [153, 194.5]	170 ± 6.08 [157, 179.5]
Weight; Kg	83 ± 15.28 [54, 114]^†^	69 ± 13.16 [47, 95]	73 ± 12.08 [58, 96]
BMI; kg/m^2^	27 ± 4.55 [19.83, 35.19]^†^	23 ± 3.14 [19.26, 29.41]	25 ± 2.86 [20.55, 30.13]
IPAQ_sit	397 ± 134.81 [180, 600]	346 ± 131.68 [180, 600]	376 ± 171.72 [180, 750]
IPAQ_walk	93 ± 53.44 [15, 180]	81 ± 60.48 [20, 180]	123 ± 62.35 [30, 240]
IPAQ_moderate	78 ± 50.23 [25, 180]	93 ± 86.58 [0, 300]	80 ± 64.72 [0, 240]
IPAQ_vigorous	66 ± 44.07 [0, 180]	52 ± 49.34 [0, 180]	50 ± 46.92 [0, 120]
UPDRS	35 ± 6.93 [24, 48]*	27 ± 7.96 [11, 39]	
Years living with PD	6.2 ± 4.13 [1.67, 12.75]	5.9 ± 4.00 [0.72, 15.90]	
Levodopa dose equivalent; mg/day	520 ± 435.88 [0, 1575]	564 ± 372.93 [0, 1200]	
Chronotropic index	0.68 ± 0.08 [0.52, 0.79]^*†^	0.97 ± 0.08 [0.85, 1.10]	1.03 ± 0.09 [0.90, 1.16]

Data is presented in mean ± SD [min, max] values. BMI: Body Max Index; IPAQ: International Physical Activity Questionnaire (sitting (sit), walking (walk), moderate, and vigorous activities); UPDRS: Unified Parkinson’s Disease Rating Scale

*p ≤ 0.05 VS control

^†^*p* ≤ 0.05 VS PD non-CI

### Physiologic outcomes

**Fig. 1:**
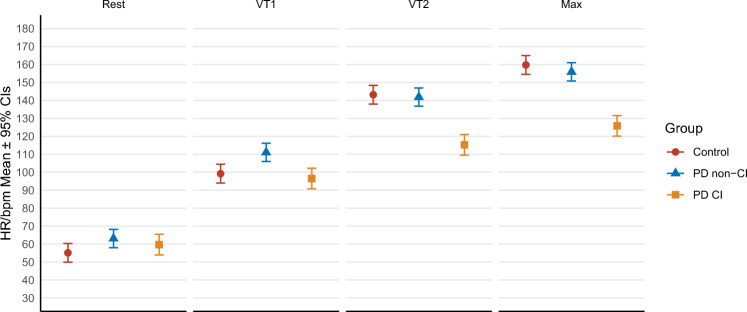
Represents heart rate (HR) responses at rest, submaximal (first (VT1) and second (VT2) ventilatory thresholds) and maximal exercise intensities. PD participants with blunted HR response (PDCI) exhibited significantly lower HR at VT2 and during peak exercise, compared to PD participants without chronotropic incompetence (PD non-CI) and controls (*p* ≤ 0.001). Differences in heart rate at VT2 (*p* = 0.738) and peak exercise (*p* = 0.332) are not significant between PD non-CI group and controls

Key-physiologic responses are presented in Table 2*.* PDCI and controls presented a similar HR at rest and at VT1 (*p* = 0.283 and *p* = 0.522). However, HR was significantly higher in PD non-CI than controls at rest (*p* = 0.045), and at VT1 compared to PDCI (*p* ≤ 0.001) and controls (*p* = 0.003). Differences in HR responses were most evident as exercise intensity increased (Fig. 1*)*. PDCI showed a significantly lower HR at VT2 and peak exercise than PD non-CI and controls (*p* ≤ 0.001). PD non-CI and controls presented a similar HR at VT2 (*p* = 0.738*)* and peak exercise (*p* = 0.332).

**Table 2:** Physiologic outcomes

	PDCI (*n* = 13)	PD non-CI (*n* = 15)	Control (*n* = 17)
Rest			
HR	60 ± 3 [54–65]	63 ± 3 [58–68]*	55 ± 3 [50–60]
SBP; mm Hg	130 ± 14 [101–159]	133 ± 8 [115–151]	124 ± 18 [86–162]
DBP; mm Hg	77 ± 6 [64–91]	79 ± 4 [71–87]	77 ± 8 [60–95]
VT1			
HR; bpm/min	96 ± 3 [91–102]^**†**^	111 ± 3 [106–116]*	99 ± 3 [94–104]
%HRmax	76 ± 3 [72–79]*^†^	70 ± 2 [67–73]*	64 ± 2 [61–68]
%MA-PHR	61 ± 1	70 ± 2	63 ± 2
*V*O_2_; ml/kg/min	14 ± 2 [11–17]	17 ± 1 [14–20]	16 ± 1 [12–19]
%*V*O_2_	61 ± 2 [56–65]*	57 ± 2 [53–61]	52 ± 2 [48–56]
WR/Kg	0.9 ± 0.1 [0.7–1.2]	1.2 ± 0.1 [0.9–1.4]	1.2 ± 0.1 [0.9–1.5]
%WR	49 ± 2 [45–53]^**†**^	44 ± 2 [40–48]	44 ± 2 [39–48]
VT2			
HR; bpm/min	115 ± 3 [109–121]*^†^	142 ± 3 [137–147]	143 ± 3 [138–148]
%HRmax	91 ± 2 [87–94]	90 ± 2 [87–93]	92 ± 2 [88–95]
%MA-PHR	73 ± 1	89 ± 2	91 ± 2
*V*O_2_; ml/kg/min	21 ± 2 [18–24]* †	27 ± 1 [24–30]	26 ± 1 [23–29]
%*V*O2	89 ± 2 [85–94]	89 ± 2 [85–93]	87 ± 2 [83–91]
WR/Kg	1.6 ± 0.1 [1.4–1.9]*^†^	2.2 ± 0.1 [2–2.5]	2.3 ± 0.1 [2–2.5]
%WR	84 ± 2 [80–88]	83 ± 2 [79–87]	82 ± 2 [78–86]
Peak exercise			
HRmax; bpm/min	126 ± 3 [120–132]*^†^	156 ± 3 [151–161]	160 ± 3 [155–165]
MA-PHR	156 ± 2	158 ± 1	158 ± 2
%MA-PHR	80 ± 1	98 ± 1	102 ± 1
*V*O_2_; ml/kg/min	24 ± 2 [21–27]*^†^	31 ± 1 [28–34]	30 ± 1 [27–33]
*V*O_2_/HR	15.4 ± 0.8 [13.7–17.1]	14.4 ± 0.7 [12.9–15.8]	13.7 ± 0.8 [12.1–15.3]
WR/Kg	2 ± 0.1 [1.7–2.3]*^†^	2.7 ± 0.1 [2.5–3]	2.7 ± 0.1 [2.5–3]
*V*O_2_/WR slope	9.1 ± 0.3 [8.5–9.8]	8.8 ± 0.3 [8.2–9.4]	9.4 ± 0.3 [8.7–10.1]
RPE	18.8 ± 0.4 [18–19.7]	19.1 ± 0.4 [18.3–19.8]	18.7 ± 0.4 [17.9–19.5]
RER	1.11 ± 0.02 [1.07–1.14]*	1.14 ± 0.02 [1.11–1.17]*	1.22 ± 0.02 [1.19–1.26]
SBP; mm Hg	174 ± 7 [160–187]	182 ± 6 [170–195]	190 ± 6 [177–203]
DBP; mm Hg	79 ± 2 [74–84]	83 ± 2 [78–87]	81 ± 2 [77–86]
Recovery			
HR1min; beats	14 ± 3 [8–20]	17 ± 3 [12–22]	20 ± 3 [15–26]
HR2min; beats	22 ± 3 [16–28]*^†^	32 ± 3 [27–37]	38 ± 3 [33–44]

All values are mean ± SE [95% CI]. BMI: Body Max Index; IPAQ: International Physical Activity Questionnaire (sitting (sit), walking (walk), moderate, and vigorous activities); UPDRS: Unified Parkinson’s Disease Rating Scale. Maximum age-predicted heart rate (MA-PHR, 220-age)

*p ≤ 0.05 VS control

^†^*p* ≤ 0.05 VS PD non-CI

**Fig. 2:**
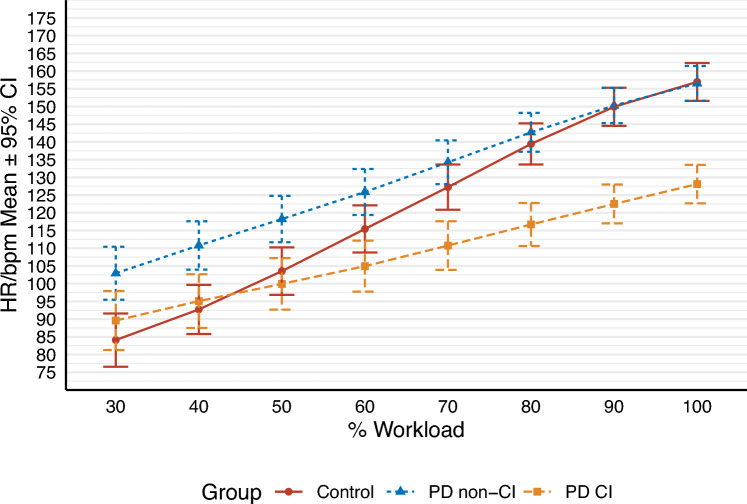
Represents changes in heart rate (HR) in beats per minute (bpm) from 30 to 100% of the maximum workload (WRmax) during the cardiopulmonary exercise test (CPET). As the workload increased, the reduced increase in heart rate responses became more apparent in PD with chronotropic incompetence (PDCI) compared to those without chronotropic incompetence (PD non-CI) and controls

When expressed as a percentage of HRmax, HR at VT1 occurred at a significantly higher relative intensity in PDCI than PD non-CI (*p* = 0.017) and controls (*p* ≤ 0.001), and in PD non-CI than controls (*p* = 0.025). The percentage of HRmax at VT2 was similar between groups (*p* ≥ 0.05). Figure 2 illustrates the dynamic changes in HR from 30% WRmax to peak exercise. PDCI had a lower HR than PD non-CI from 30% WRmax to 100% WRmax (*p* ≤ 0.05). HR responses of PDCI were similar to those of control participants up to 50% WRmax (*p* ≥ 0.05); however, the increase in HR between 50 and 100% WRmax was significantly lower in PDCI (mean of 28 bpm) compared to control subjects (mean of 53 bpm) (*p* ≤ 0.05). At peak exercise, PDCI had a significantly lower HR than PD non-CI and control groups (*p* ≤ 0.001), which had similar HRs (*p* = 0.915). Recovery HR following CPET was similar in the first minute among groups (*p* ≥ 0.05), but HR2min was significantly lower in PDCI than PD non-CI (*p* = 0.011) and controls (*p* ≤ 0.001). PD non-CI and controls presented a similar HR2min (*p* = 0.107).

**Fig. 3:**
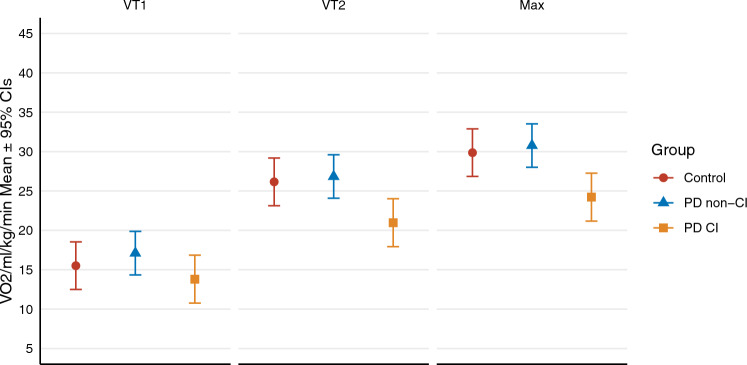
Represents the difference in oxygen consumption (*V*O_2_) at submaximal (first (VT1) and second (VT2) ventilatory thresholds) and maximal exercise intensities between groups. PD participants with blunted HR response (PDCI) presented significantly lower *V*O_2_ compared to both PD participants without chronotropic incompetence (PD non-CI) and the control group at VT2 (*p* = 0.003 and *p* = 0.036) and peak exercise (*p* = 0.001 and *p* = 0.023)

*V*O_2_ and WR were similar among groups at VT1 (*p* ≥ 0.05). Data illustrated in Fig. 3 shows that *V*O_2_ was significantly lower in PDCI than PD non-CI and controls at VT2 (*p* = 0.003 and *p* = 0.036) and peak exercise (*p* = 0.001 and *p* = 0.023). WR/kg was also significantly lower in PDCI than PD non-CI and controls at VT2 (*p* = 0.002 and *p* = 0.008) and peak (*p* ≤ 0.001 and *p* = 0.002). The *V*O_2_/WR slope, *V*O_2_/HR, and blood pressure (SBP and DBP) at peak exercise were similar among groups (*p* ≥ 0.05).

When expressed as a percentage of % *V*O_2_ peak, oxygen consumption at VT1 was significantly higher in PDCI than controls (*p* = 0.014), while no significant differences were detected at VT2 among groups (*p* ≥ 0.05). %WRmax attained at VT1 was significantly higher in PDCI than PD non-CI (*p* = 0.049), but similar among groups at VT2 (*p* ≥ 0.05). RPE was not significantly different (*p* ≥ 0.05) between groups at maximal exercise intensity. The most reported symptom-limiting factor at the end of the tests by all participants was leg muscle fatigue (PDCI *n* = 11 (85%) PD non-CI *n* = 12 (80%), and controls *n* = 12 (71%)).

## Discussion

To our knowledge, this is the first study to examine HR and metabolic responses to an incremental increase in workload in a group of people with PD classified according to the presence of CI. Our key finding was that people with PD with CI had blunted HR responses which were evident at high (VT2) and maximal intensity exercise but not at rest or during moderate intensity (VT1). This brings into question the accuracy of age-predicted equations for exercise intensity prescription for this subgroup of PD. Our secondary finding was that this subgroup also presented lower aerobic capacity at VT2, and peak exercise compared to PD non-CI and controls.

Our findings show that CI was present in 46.4% of PD (H&Y 1–3) participants, in line with previous studies reporting a prevalence of 40–62.2% (H&Y 2–3), and, comparable to our results, also showing an inability to reach 85% of their age-predicted maximum HR (Bryant et al. 2016; Penko et al. 2021; Werner et al. 2006). These results differ from Katzel et al. (2011) who reported that only 7 (11%) of 63 participants with PD (H&Y 1.5–3) were able to achieve 85% of their age-predicted maximum HR. Contrasting findings may be due to differences in CPET test modality and criteria for attainment of maximum effort. Participants from Katzel et al. (2011) were tested on the treadmill and attained an RER below 1.05, while participants from our study were tested on the cycle ergometer and presented an RER above the recommended threshold of 1.10 (Robergs et al. 2010).

PDCI exhibited significantly lower changes in HR compared to other groups at higher workload intensities despite having a similar HR at rest and moderate intensity (VT1) compared to controls. Werner et al. (2006) also reported similar HR at rest and at moderate intensities (stage 2 of the Modified Bruce Protocol), and lower HR at the termination of the test in a subgroup of PD who were not able to attain 85% of their age-predicted maximum HR compared to controls. Evaluation of heart rate variability (HRV) was beyond the scope of this study, but our own findings and those from the literature suggest that the mechanisms contributing to a slow rate of change in HR in PD are likely to be linked to a reduced-sympathetic nervous system (SNS) drive. In healthy individuals, at high intensities, an increase in HR is primarily driven by the SNS yielding a rise in norepinephrine (DiFrancisco-Donoghue et al. 2009; White and Raven 2014). This contrasts with lower levels of norepinephrine, which is also associated with CI (Grosman-Rimon et al. 2023), found at peak exercise in people with PD both on and off medication (DiFrancisco-Donoghue et al. 2009).

Oxygen consumption at VT2 and peak exercise were lower in PDCI compared to PD non-CI and controls, whereas no significant differences were found between PD non-CI and controls. These findings are difficult to compare with earlier studies because PD participants are not usually stratified according to CI. However, it may help explain earlier ambiguous findings, with some studies reporting lower levels of *V*O_2_ peak in PD compared with controls (Kanegusuku et al. 2016; Katzel et al. 2011; Mavrommati et al. 2017) and others not (DiFrancisco-Donoghue et al. 2009; Protas et al. 1996). The mechanisms causing reduced aerobic capacity in PD with CI are not well understood. A blunted HR response which reduces maximal cardiac output, and malfunctioning of the mitochondria which reduces the arterio-venous oxygen difference (Larsen et al. 2020; Liguori et al. 2022) are both potential factors compromising aerobic capacity in PD (DiFrancisco-Donoghue et al. 2009; Kanegusuku et al. 2016; Penko et al. 2021). Although arterio-venous oxygen difference was not evaluated in this study, our results suggest that failure to achieve the age-predicted maximum HR may be one of the primary factors influencing *V*O_2_ peak in PDCI, given that the *V*O_2_/WR slope and oxygen pulse (*V*O_2_/HR) at peak exercise were similar between groups. *V*O_2_/HR is an indirect measure of stroke volume and in addition to the *V*O_2_/WR slope, indicates that PDCI participants were able to efficiently extract oxygen per heartbeat and per unit of work but unable to achieve their age-predicted maximum HR and associated cardiac output at maximal effort (Wasserman 2012).

Similar IPAQ results between PD and control subjects suggest that all participants were physically active and met the minimum recommendations for aerobic training from the ACSM (Liguori et al. 2022). However, the mean *V*O_2_ peak in the PDCI was on average 5 ml.kg-1 lower than control subjects. *V*O_2_ peak is an independent predictor of mortality, and a decrease in 1 metabolic equivalent of task (3.5 ml.kg-1) is associated with an increased risk of morbidity and mortality (Ezzatvar et al. 2021). By any means, the *V*O_2_ peak for PDCI was considerably higher than earlier reports (DiFrancisco-Donoghue et al. 2009; Kanegusuku et al. 2016; Penko et al. 2021), suggesting that involvement in high levels of training may be beneficial for PD but still not enough for PDCI to achieve similar levels of fitness. Improvement in *V*O_2_ peak is dependent on a variety of biologic and methodological factors (Meyler et al. 2021), limiting the scope of interpretation. Although, blunted-HR has previously been associated with reduced improvement in *V*O_2_ peak in PD (Penko et al. 2021) and in other clinical populations (Herbsleb et al. 2019), further studies evaluating the effect of aerobic training in PD with CI are required.

Based on our data, to achieve the threshold that represents moderate intensity (VT1), PD with CI need to train at a significantly higher percentage of their HRmax than PD non-CI and control subjects. These findings are consistent with earlier work, for example participants with CVD and a blunted heart rate response achieved 75–85% of their HRmax at VT1 during CPET (Anselmi et al. 2021; Smarz et al. 2021), comparable to our findings. We used CPET to examine response to aerobic exercise and stratify according to CI. Aerobic protocols that use the threshold zone derived from CPET to set and monitor exercise intensity are more accurate than those relying on predicted equations (de Lira et al. 2017; Meyler et al. 2021; Pymer et al. 2020). However, this approach is not readily available to clinicians who instead approximate values.

Alberts and Rosenfeldt (2020) recommend an intensity of 70–85% of HRmax or an RPE of 14–17 for aerobic training due to the high prevalence of ANS dysfunction in this population, whereas the ACSM recommends a progressive increase in exercise intensity from 60–65% to 80–85% HRmax (Liguori et al. 2022). Our results suggest greater accuracy may be obtained using an RPE of ≥ 18 to indicate that maximum effort has been achieved (Liguori et al. 2022). From this, a more accurate estimate of HRmax can be obtained rather than using predicting equations.

## Conclusion

CI is common in people with PD, attenuating HR and metabolic responses at high and peak exercise. It must therefore be considered when establishing aerobic exercise protocols for PD. In clinical practice, an RPE > 18 may provide a more precise estimate of HRmax than the use of predicted equations. Future research is required to understand the effect of CI on improving aerobic capacity and its association with disease progression, CVD, and other manifestations of ANS dysfunction.

## Study limitations

Participants from this study were classified with mild disease severity (H&Y 1–3), limiting generalizability. We used a bicycle ramp incremental protocol which may yield different results compared with other protocols and types of equipment. Although HRV was not analyzed in this study, the assessment of this sensitive measure warrants further investigation in PwPD with and without CI. Finally, although adequately powered for the primary outcome, the sample size was small, and our results are therefore exploratory.

## Data Availability

The data supporting the results of this study can be obtained by contacting the corresponding author upon reasonable request.
